# Generation and miRNA Characterization of Equine Induced Pluripotent Stem Cells Derived from Fetal and Adult Multipotent Tissues

**DOI:** 10.1155/2019/1393791

**Published:** 2019-05-02

**Authors:** Laís Vicari de Figueiredo Pessôa, Pedro Ratto Lisboa Pires, Maite del Collado, Naira Caroline Godoy Pieri, Kaiana Recchia, Aline Fernanda Souza, Felipe Perecin, Juliano Coelho da Silveira, André Furugen Cesar de Andrade, Carlos Eduardo Ambrosio, Fabiana Fernandes Bressan, Flavio Vieira Meirelles

**Affiliations:** ^1^Departamento de Medicina Veterinária, Faculdade de Zootecnia e Engenharia de Alimentos, Universidade de São Paulo, Pirassununga 13635-000, Brazil; ^2^Department of Veterinary and Animal Sciences, Section for Anatomy & Biochemistry, University of Copenhagen, 1870 Frederiksberg C, Denmark; ^3^Departamento de Reprodução Animal, Faculdade de Medicina Veterinária e Zootecnia, Universidade de São Paulo, Pirassununga 13635-000, Brazil

## Abstract

**Introduction:**

Pluripotent stem cells are believed to have greater clinical potential than mesenchymal stem cells due to their ability to differentiate into almost any cell type of an organism, and since 2006, the generation of patient-specific induced pluripotent stem cells (iPSCs) has become possible in multiple species.

**Objectives:**

We hypothesize that different cell types respond differently to the reprogramming process; thus, the goals of this study were to isolate and characterize equine adult and fetal cells and induce these cells to pluripotency for future regenerative and translational purposes.

**Methods:**

Adult equine fibroblasts (eFibros) and mesenchymal cells derived from the bone marrow (eBMmsc), adipose tissue (eADmsc), and umbilical cord tissue (eUCmsc) were isolated, their multipotency was characterized, and the cells were induced *in vitro* into pluripotency (eiPSCs). eiPSCs were generated through a lentiviral system using the factors OCT4, SOX2, c-MYC, and KLF4. The morphology and *in vitro* pluripotency maintenance potential (alkaline phosphatase detection, embryoid body formation, *in vitro* spontaneous differentiation, and expression of pluripotency markers) of the eiPSCs were characterized. Additionally, a miRNA profile analysis of the mesenchymal and eiPSCs was performed.

**Results:**

Multipotent cells were successfully isolated, but the eBMmsc failed to generate eiPSCs. The eADmsc-, eUCmsc-, and eFibros-derived iPSCs were positive for alkaline phosphatase, OCT4 and NANOG, were exclusively dependent on bFGF, and formed embryoid bodies. The miRNA profile revealed a segregated pattern between the eiPSCs and multipotent controls: the levels of miR-302/367 and the miR-92 family were increased in the eiPSCs, while the levels of miR-23, miR-27, and miR-30, as well as the let-7 family were increased in the nonpluripotent cells.

**Conclusions:**

We were able to generate bFGF-dependent iPSCs from eADmsc, eUCmsc, and eFibros with human OSKM, and the miRNA profile revealed that clonal lines may respond differently to the reprogramming process.

## 1. Introduction

Mesenchymal stem cells (MSCs) are spindle-shaped multipotent cells that are easy to isolate, readily adhere to plastic culture dishes, and have great expansion capacity. MSCs have been widely used for cell therapy over the last few years despite their rare presence in tissues and their limited differentiation potential [1–3]. Due to their ability to differentiate into almost any cell type of an organism, pluripotent stem cells, such as embryonic stem cells (ESCs), are highly significant and efficiently isolated and maintained *in vitro* in domestic species [4, 5]. Since Yamanaka's breakthrough in 2006, it has been possible to produce patient-specific induced pluripotent stem cells (iPSCs), which are currently available for multiple species, including horses [6–11].

iPSCs hold great potential for use in both human and veterinary regenerative medicine. The influence of the origin of the somatic cells used in iPSC production is currently controversial. Equine iPSCs have already been produced from fibroblasts [6–10] and mesenchymal cells derived from adipose tissue [11].

Previous reports have suggested that iPSCs may retain residual epigenetic traces of the cell types from which they were reprogrammed, which could affect the reprogramming and differentiation capacity of these cells [12]. Additionally, it has been reported that fibroblasts present inferior reprogramming rates compared with other cell types [13]. Therefore, studies investigating multiple cell sources from the same individuals are particularly important for enhancing our understanding and use of these cells for further applications.

Because of the more than 100 billion dollars invested in horses per year [10], the severe economic losses caused by horse musculoskeletal injuries [7], and due to the similarities between human and horse athletes and between injuries resulting from physical activity in both species [14], horses can be considered excellent models for musculoskeletal research and cell therapy.

It has been shown that miRNAs are relevant to the regulation of reprogramming and the maintenance of pluripotency and may present different expression patterns according to the different pluripotency states [15]. Some miRNA families, such as miR-302/367 and miR-92, present increased levels in iPSCs and ESCs, while differentiated cells present increased levels of miR-23, miR-27, and miR-30, as well as the let-7 family [16, 17]. Thus, miRNA profile analyses may provide important information regarding pluripotency induction, specifically in those species for which iPSC generation is not fully understood.

Herein, we aimed to obtain and characterize the following fetal and adult equine cell lines: equine umbilical cord tissue mesenchymal cells (eUCmsc), adipose tissue mesenchymal cells (eADmsc), bone marrow mesenchymal cells (eBMmsc), and equine adult fibroblasts (eFibros). These cells were subsequently characterized and induced into pluripotency to generate eiPSCs (eiPSCs-eADmsc, eiPSCs-eUCmsc, eiPSCs-eBMmsc, and eiPSCs-eFibros) using human or murine *OCT4*, *SOX2*, *KLF4*, and *C-MYC* (hOSKM and mOSKM, respectively). The generated eiPSCs were maintained for more than 30 passages only on bFGF supplementation, and their morphology, *in vitro* pluripotency maintenance potential (as measured by alkaline phosphatase detection), *in vitro* spontaneous differentiation, and pluripotency-related factor expression (as measured by immunocytochemistry and expression of transcripts) were analyzed. Additionally, compared to other cell types, the miRNA profile of the multipotent and eiPSCs produced here suggests that a heterogeneous reprogramming process occurred.

## 2. Methods

The study procedures were performed after obtaining approval from the Ethics Committee on the Use of Animals of the Faculty of Veterinary Medicine and Animal Sciences, University of São Paulo (protocol 2913/2013). Unless otherwise stated, the analyses were performed in biological triplicates, and photodocumentation was performed with a Nikon Eclipse TS100 and Nikon DS-Ri1.

### 2.1. Cell Line Isolation and Characterization

At least three cell lines were isolated from different animals per cell type. The cells were initially cultivated in 35 mm diameter dishes using Iscove's Modified Dulbecco's Medium (IMDM, Life Technologies) supplemented with 15% fetal bovine serum (FBS, Gibco), 0.1 mg/ml streptomycin, and 100 U/ml penicillin (pen/strep, Gibco) at 38.5°C, 5% CO_2_, and maximal humidity (standard culture conditions), unless otherwise stated.

#### 2.1.1. Bone Marrow Mesenchymal Stem Cells

The equine bone marrow cells were provided by Dr. Fernanda da Cruz Landim (São Paulo State University, UNESP, Botucatu, SP, Brazil) and isolated as previously described [18].

#### 2.1.2. Fibroblasts and Adipose Tissue Mesenchymal Stem Cells

The fibroblasts were obtained from skin fragments from adult mares' necks, and the adipose tissue mesenchymal cell lineages were obtained from biopsies of adipose tissue removed from the tail ply. The collected skin fragments and adipose tissue biopsies were rinsed in a 0.9% saline solution containing antibiotics, minced to small pieces, and digested in 5 ml of collagenase IV 0.001% (Sigma-Aldrich) for 3 h at 38.5°C. Next, the collagenase was inactivated, and the cell precipitate was suspended in IMDM media and cultured under standard culture conditions. The cells were passaged before reaching 90% confluency and cryopreserved for further studies.

#### 2.1.3. Umbilical Cord Tissue Mesenchymal Stem Cells

The eUCmsc were derived from an umbilical cord fragment collected postpartum immediately after the natural rupture of the cord. The tissue was rinsed in a 0.9% saline solution containing antibiotics, reduced into small fragments before incubation, cultured in IMDM medium under standard culture conditions, passaged before reaching 90% confluency, and cryopreserved after 48 h in culture for further studies.

#### 2.1.4. Cell Characterization by Multilineage Differentiation

The cells were subjected to induced differentiation using commercial kits (StemPro A10070-01, A10071-01, and A10072-01; Gibco) according to the manufacturer's instructions. Briefly, for the chondrogenic cell differentiation, the eBMmsc, eADmsc, and eUCmsc were plated in 5 *μ*l droplets at a concentration of 1.6 × 10^7^ cells/ml and cultured under standard conditions for 2 h; then, the differentiation medium was added. The medium was replaced every 4 days for 2 weeks. For the osteogenic differentiation, 5 × 10^3^ cells/cm^2^ were plated and incubated for 24 h; then, the IMDM medium was replaced by a specific differentiation medium, which was replaced every 4 days for 2 weeks. For the adipogenic differentiation, 1 × 10^4^ cells/cm^2^ were plated and incubated for 24 h; then, the IMDM medium was replaced by a specific differentiation medium, which was replaced every four days for two weeks. The cells in the control group were plated under the same conditions but were maintained in IMDM medium. At the end of the two-week period, the cells were fixed and stained using Sudan black dye (Sigma-Aldrich) for the adipogenesis differentiation detection, Alizarin red (Sigma-Aldrich) for the osteogenesis detection, and Alcian blue (Sigma-Aldrich) for the chondrogenesis differentiation detection.

#### 2.1.5. Analysis of Surface Markers on Mesenchymal Stem Cells

The equine specific surface markers CD44 (MCA1082GA, AbD Serotec, Raleigh, NC, USA), CD86 (NB100-77815, BD Pharmingen), MHC I (MCA1086GA, AbD Serotec), and MHC II (MCA1085GA, AbD Serotec) were analyzed by flow cytometry (FACSAria, BD Biosciences) as previously described [19–21]. In brief, the eADmsc, eUCmsc, eBMmsc, eFibros, and MEFs (which were used as a negative control) were pelletized, rinsed with 0.1% BSA solution, and centrifuged at 600*g* for 5 min (min). Subsequently, the cells were blocked for 1 h with 10% bovine serum albumin (BSA, Sigma-Aldrich) solution and rinsed. Then, the cells were incubated with primary antibodies (dilution 1 : 300) for 1 h, rinsed, and pelletized again (600*g*, 5 min). The secondary antibody (polyclonal goat anti-mouse FITC; dilution 1 : 300, F-0479, Dako) was added for 1 h; then, the cells were rinsed with 2% paraformaldehyde (PFA, Synth) solution for 10 min and suspended in phosphate-buffered saline (PBS, Sigma-Aldrich).

The analysis of the fluorescence was performed using a FACSAria cytometer (BD Biosciences) controlled by FACSDiva v.8 software (BD Biosciences). At least 1 × 10^5^ cells per group were analyzed to determine their size and complexity (FSC × SSC) and FITC fluorescence intensity (excitation 488 nm, emission 530 nm).

#### 2.1.6. Analysis of Cellular Doubling Time

The cell lines were plated in 6-well plates at 3 × 10^4^ cells/well. After 48 h in culture, the cells were counted with a Neubauer chamber and replated at the initial ratio. This process was repeated five times. The mean cell doubling time (DT) was calculated in hours as previously described [22] using the equation DT = (*T* − *T*0) log 2/(log *N* − log *N*0), where (*T* − *T*0) is the time (hours) during which the cells were cultured between passages, *N*0 is the number of cells originally plated, and *N* is the number of cells harvested after 48 h of culture.

### 2.2. Cellular Reprogramming

#### 2.2.1. Induction of Pluripotency

The eADmsc, eUCmsc, eFibros, and eBMmsc were induced to pluripotency using murine or human *OCT4*, *SOX2*, *KLF4*, and *C-MYC* (mOSKM and hOSKM, respectively) reprogramming factors in a lentiviral vector (STEMCCA, Millipore) [23]. Lipofection (Lipofectamine 3000, Life Technologies) was used for the lentiviral production. In total, 6 × 10^6^ 293FT cells were plated in 100 mm diameter dishes one day before lipofection to achieve 90% confluency at the time of transfection. Twelve micrograms of each OSKM vector, 1.2 *μ*g of each auxiliary vectors, i.e., TAT, REV, and Hgpm2, and 2.4 *μ*g of VSVG were used in the transfection per plate. The cells were incubated with lipofection overnight, and the culture medium was collected after 24, 48, and 72 h, filtrated, and used for the transduction.

For the transduction, 2 × 10^4^ equine cells were previously seeded in each well of a 6-well plate and transduced with hOSKM or mOSKM in the presence of 8 *μ*g/ml Polybrene (hexadimethrine bromide, Sigma-Aldrich). After six days of culture in IMDM, the cells were harvested, plated onto feeder layers (1 × 10^5^ mitomycinized mouse embryonic fibroblasts (MEFs) on 6-well plates), and cultivated in KnockOut Dulbecco's modified Eagle's medium (DMEM/F-12, Gibco), 10% KnockOut Serum Replacement (KSR, Gibco), 1% antibiotics (pen/strep, Gibco), 1% L-glutamine (Gibco), 1% nonessential amino acids (Gibco), and 0.007% *β*-mercaptoethanol (Gibco) supplemented with 10 ng/mL human bFGF (PeproTech) until colonies formed. The first passage of each culture was performed manually for the clonal iPSC lineage formation; then, dissociation (TrypLE Express, Gibco) was used for replating.

#### 2.2.2. Characterization of iPSCs: Alkaline Phosphatase Detection, Embryoid Body (EB) Formation, Spontaneous Differentiation *In Vitro*, and Immunocytochemical Analysis

Alkaline phosphatase (AP) was detected in iPSCs using an Alkaline Phosphatase Detection Kit (86R-1KT, Sigma-Aldrich) according to the manufacturer's instructions. For the EB formation, eiPSCs were dissociated (TrypLE Express, Gibco) for 2 min, and the cell clumps were plated in culture dishes (previously coated with 0.6% agarose to avoid adhesion) along with iPSC culture medium without bFGF. The EBs were cultivated for three to five days; then, they were mechanically dissociated and plated in IMDM and the cells were cultured for 30 days for spontaneous differentiation.

Immunocytochemical analysis was performed on the cells to detect ectoderm tissue formation through neurofilament labeling. The cells were fixed with 4% PFA for 12 min in the wells and washed with PBS 3 times for 5 min per wash. Then, the cells were permeabilized with 0.1% Triton X-100 (Labome) solution for 10 min at room temperature, washed with PBS 3 times for 5 min per wash, and blocked with 1% BSA/0.1% Tween 20 (Biocare Medical) solution for 30 min at room temperature. Then, the cells were labeled with the primary antibody (Neurofilament, N4142, Sigma-Aldrich; 1 : 100 dilution) overnight, washed with PBS 3 times, and labeled with secondary antibodies (anti-rabbit 488, A11034, Invitrogen; 1 : 300 dilution) for 1 h.

#### 2.2.3. OCT4 Immunocytochemical Analyses in 24-Well Plates

The eiPSCs were cultured in the proper medium until colonies were apparent; then, the cells were fixed, permeabilized, blocked, and labeled with a primary OCT4 antibody (anti-goat Sc8628, Santa Cruz Biotechnology; 1 : 100 dilution) and secondary antibody (anti-goat Alexa Fluor 488, A11078; 1 : 300 dilution, Invitrogen) as described above. The cells were washed 3 times with PBS for 5 min per wash, labeled with Hoechst 33342 (1 : 1000 dilution, Sigma-Aldrich) for 5 min, and washed again with PBS. In both analyses, noninduced cells were used as negative controls.

#### 2.2.4. Detection of Transcripts Related to Pluripotency and Reprogramming Vectors

For the total RNA extraction, the cells were pelleted at passages 5-7 by centrifugation, frozen in liquid nitrogen, and stored in a freezer at -80°C. The RNA was isolated with the TRIzol Reagent (Invitrogen), and the extraction was carried out using a miRNeasy Mini Kit (Qiagen) according to the manufacturer's instructions. After the DNase treatment, a NanoDrop 2000 (Thermo Fisher Scientific) was used to measure the total RNA, and the RNA quality was evaluated by the 260/280 ratio. The cDNA synthesis was performed with a high-capacity cDNA reverse transcription kit (Applied Biosystems) according to the manufacturer's instructions.

A conventional PCR analysis was performed to detect the expression of genes related to pluripotency (*NANOG* and *OCT4*) and the exogenous reprogramming vector (Table 1). *NANOG* was analyzed since it is not included in the reprogramming vector; therefore, its presence, which is exclusively endogenous, may be considered an indicator of reprogramming. *OCT4* was also analyzed because of its importance for the reprogramming process and because of its expression from both endogenous and exogenous cDNAs.

To analyze the transcripts related to pluripotency, we performed a 25 *μ*l PCR reaction containing 12.5 *μ*l of 2x Master Mix (BioLab), 200 nM of specific reverse primer and forward primer, 1 *μ*l of cDNA, and diethyl pyrocarbonate (DEPC) water in a final volume of 25 *μ*l. The PCR reaction was performed under the following cycle conditions: 95°C for 1 min; 35 cycles of 95°C for 30 seconds (s), 57.5°C for 1 min, and 68°C for 1 min; and finally, 68°C for another 5 min; the amplicon was confirmed by agarose gel (1.5%) electrophoresis. Additionally, to detect the presence of the reprogramming vector, DNA was isolated using a commercial DNeasy Blood and Tissue Kit (Qiagen). The PCR reaction was performed using the same concentrations and volumes used for the *OCT4* and *NANOG* PCR reactions; however, the annealing temperature was 60°C. *GAPDH* was used as a housekeeping gene for the vector integration and expression, and eADmsc, eUCmsc, and eFibros were used as negative controls for the *NANOG* and *OCT4* detection. The amplicon was confirmed by agarose gel electrophoresis.

### 2.3. miRNA Profile Analysis

The miRNA expression profiles of eFibros, eADmsc, eUCmsc, and their derived iPSCs, as well as the MEFs used for the iPSC culture were analyzed. The RNA extraction was performed with a miRNeasy Mini Kit (Qiagen). A miScript PCR System (Qiagen) was used for the miRNA reverse transcription according to the manufacturer's instructions. Then, 100 ng of total RNA was converted by RT-PCR to cDNA and used for the expression analyses of 126 miRNAs (124 target miRNAs and 2 housekeeping genes, Supplementary material 1). Briefly, the RNA was incubated with 5x miScript HiFlex Buffer, 10x miScript Nucleic Mix, RNase-free water, and miScript Reverse Transcriptase at 37°C for 60 min, followed by 5 min at 95°C. An RT-qPCR reaction was set up in a volume of 10 *μ*l containing 2x QuantiTect SYBR Green (Qiagen), 10 *μ*M each of a universal reverse primer (Qiagen) and miRNA-specific forward primer, and 0.03 *μ*l of 1 : 4 diluted cDNA, and the qPCR reaction was carried out using a QuantStudio 6 Flex PCR System (Applied Biosystems) under the following cycle conditions: 95°C for 15 min; 45 cycles of 94°C for 10 s, 55°C for 30 s, and 72°C for 30 s. The amplicon was confirmed by a melting curve analysis. The Ct values were normalized to the geometric mean of RNT43 snoRNA, Hm/Ms/Rt T1 snRNA, and bta-mir-99b. After normalizing the data using the geometric mean of three miRNAs detected in the groups, the cycle thresholds and transcript levels were calculated using the 2^-∆Ct^ method [24]. After evaluating homology among human and equine miRNAs, the software mirPath v.3 (DIANA TOOLS) was used to identify the pathways related to the miRNAs found in each group.

### 2.4. Statistical Analysis

Statistical analyses of cellular doubling time, surface markers, and colony production efficiency were carried out using an analysis of variance, followed by a Tukey test at a significance level of 5%. The results are expressed as the means ± standard deviation.

For miRNA profile analyses, comparisons between control and treated cells for each group (eUCmsc, eFibros, and eADmsc) were made by a two-tailed Student *t*-test following two-sample and homoscedastic parameters, at a significance level of 5%. The results are expressed as the means ± standard deviation. The heat map and principal component analysis were performed after the normalized CT values (2^-∆Ct^) of all 110 miRNA commonly detected in all three cell types that were exported and processed using Metaboanalyst 3.0 (http://www.metaboanalyst.ca) [25].

## 3. Results

### 3.1. Cell Line Isolation and Characterization

#### 3.1.1. Multilineage Differentiation

**Figure 1:**
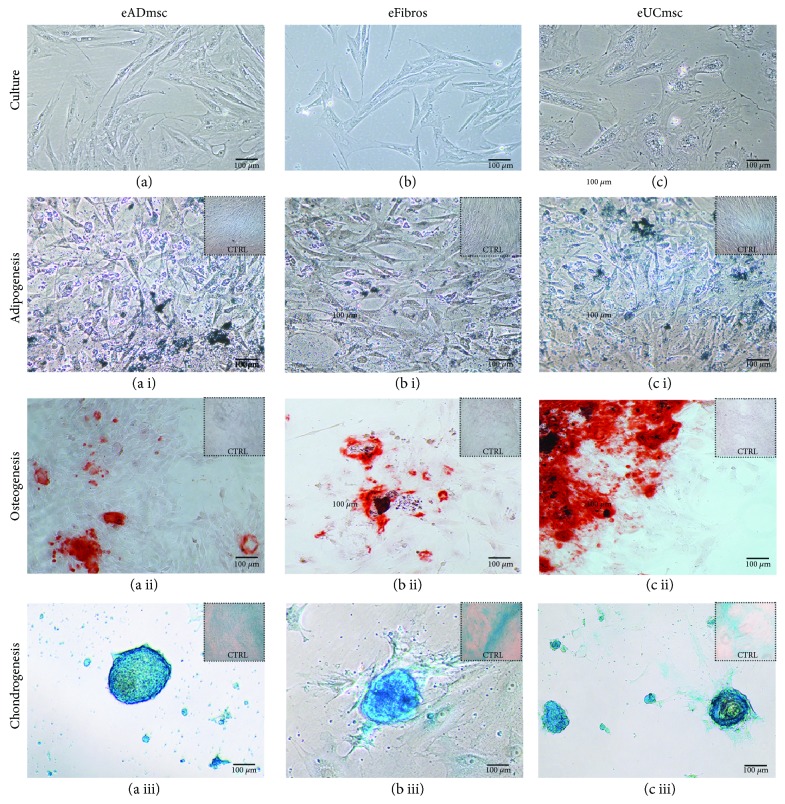
(a) Adipose tissue mesenchymal cells, (b) fibroblasts, and (c) umbilical cord tissue mesenchymal cells, 200x. After multilineage differentiation, it is possible to observe (a i, b i, and c i) adipogenic differentiated cells characterized by Sudan black-stained lipid vacuole accumulation, 200x; (a ii, b ii, and c ii) osteogenic differentiated cells characterized by calcium deposition, which were stained with alizarin red, 100x; and (a iii, b iii, and c iii) chondrogenic differentiated cells characterized by chondrogenic pellet development, which were stained with Alcian blue, 200x. Negative control cells maintained the typical spindle-like shape and differed from the treated cells.

Three eADmsc, 3 eFibros, 3 eUCmsc (Figures 1(a), 1(b), and 1(c), respectively), and 3 eBMmsc (Supplementary material 2) cell lines were successfully isolated, and their induced differentiation as indicated by morphological differences between the differentiated cells and negative control cells was analyzed. Adipogenic differentiated cells were characterized by lipid vacuole accumulation (Figures 1(a i), 1(b i), and 1(c i)), osteogenic differentiated cells were characterized by calcium deposition (Figures 1(a ii), 1(b ii), and 1(c ii)), and chondrogenic differentiated cells were characterized by chondrogenic pellet development (Figures 1(a iii), 1(b iii), and1(c iii)), while the negative control cells maintained the typical spindle-like shape.

#### 3.1.2. Analysis of Surface Markers on Mesenchymal Cells

The cell lineages presented high percentages of expression of CD44 (eADmsc, 92.5 ± 11.9; eUCmsc, 93.4 ± 5.1; eBMmsc, 86 ± 15.1; and eFibros, 89.7 ± 7.5) and MHC I (eADmsc, 84 ± 7.5; eUCmsc, 81.2 ± 17.8; eBMmsc, 61 ± 4.5; and eFibros, 94.13 ± 7.52) and low percentages of expression of CD86 (eADmsc, 1.8 ± 1.3; eUCmsc, 1.2 ± 0.7; eBMmsc, 6.4 ± 5.1; and eFibros, 0.6 ± 0.1) and MHC II (eADmsc, 3 ± 0.5; eUCmsc, 5.4 ± 1.9; eBMmsc, 13.2 ± 10; and eFibros, 5.7 ± 1.6—Table 2). No significantly different results were detected, which is consistent with the already described data of the equine surface markers.

#### 3.1.3. Analysis of Cellular Doubling Time

The doubling time assay was determined in hours, and the following results were obtained: 58 ± 14 h for eBMmsc, 46 ± 12 h for eUCmsc, 29 ± 9 h for eFIBROs, and 23 ± 11 h for eADmsc (Supplementary material 3). eADmsc and eFibros presented a significantly lower doubling time compared to eUCmsc and eBMmsc (*P* < 0.05).

### 3.2. Cellular Reprogramming

#### 3.2.1. Induction of Pluripotency

**Figure 2:**
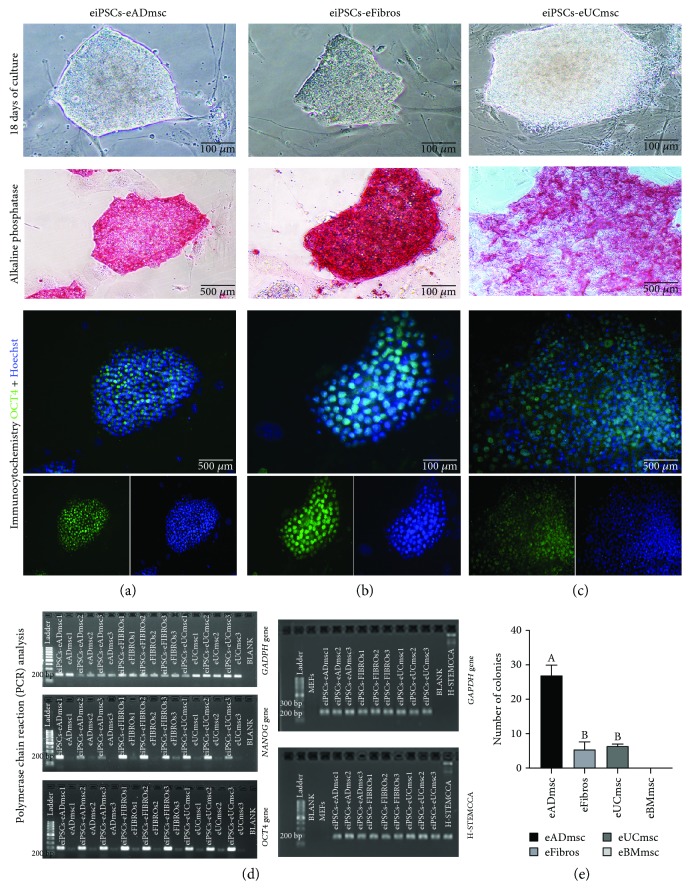
Equine iPSCs on day 18 after transduction from (a) adipose tissue mesenchymal cells, (b) fibroblasts, and (c) umbilical cord tissue mesenchymal cells, 200x. Alkaline phosphatase-positive equine iPSC colonies were induced from each cell type: (a) adipose tissue mesenchymal cells, 100x; (b) fibroblasts, 200x; (c) umbilical cord tissue mesenchymal cells, 100x. In addition, images present the immunocytochemistry expression of merged OCT4, OCT4/FITC, and Hoechst staining: (a) adipose tissue mesenchymal cells, 100x; (b) fibroblasts, 200x; (c) umbilical cord tissue mesenchymal cells, 100x. (d) Transcript expression of GAPDH, NANOG, and OCT4 in equine adipose tissue mesenchymal cells, umbilical cord tissue mesenchymal cells, and fibroblasts before and after pluripotency induction. NANOG and OCT4 expression levels are enhanced after cell reprogramming. Also in (d), confirmation of GAPDH and STEMCCA expression in equine iPSCs by conventional PCR. (e) Graph showing the total production of eiPSC colonies using hOSKM. No colonies were formed from the bone marrow mesenchymal cells. Different letters indicate significantly different results (*P* < 0.05).

The equine cells were induced into pluripotency using human or murine OKSM. Using hOSKM (Figure 2), eUCmsc were the first lineage to present iPSC colony formation (11 days after pluripotency induction), followed by eADmsc (13 days after pluripotency induction), and finally, eFibros (15 days after pluripotency induction). Using mOSKM, only eADmsc presented colony formation in one repetition; however, this result was not reproducible (*P* < 0.05) (Figure 2(e)). It was not possible to produce eiPSCs from eBMmsc in the present study using either of the reprogramming systems (Supplementary material 2).

In the comparison of the eiPSC production efficiency among the different lineages produced here, using 24 × 10^4^ cells induced to pluripotency, eiPSCs-eADmsc (*n* = 322, *P* < 0.01) presented the highest colony formation, followed by eiPSCs-eFibros (*n* = 65) and eiPSCs-eUCmsc (*n* = 58); the final two lineages did not differ (*P* = 0.95, Table 3).

In total, 85 iPS clonal cell lines were generated, further maintained *in vitro* at initial passages, and cryopreserved (human reprogramming factors: *n* = 30 for eiPSCs-eFibros, *n* = 33 for eiPSCs-eADmsc, and *n* = 21 for eiPSCs-eUCmsc; mouse reprogramming factors: n = 1 for eiPSCs-eADmsc) (Table 3), suggesting that human reprogramming factors are more effective in reprogramming equine cells in this study. At least one clonal eiPCS line from each group was maintained *in vitro* for at least 30 passages. Additionally, the equine iPSCs produced here are dependent only on bFGF, dismissing the need for LIF supplementation.

#### 3.2.2. eiPSC Characterization

**Figure 3:**
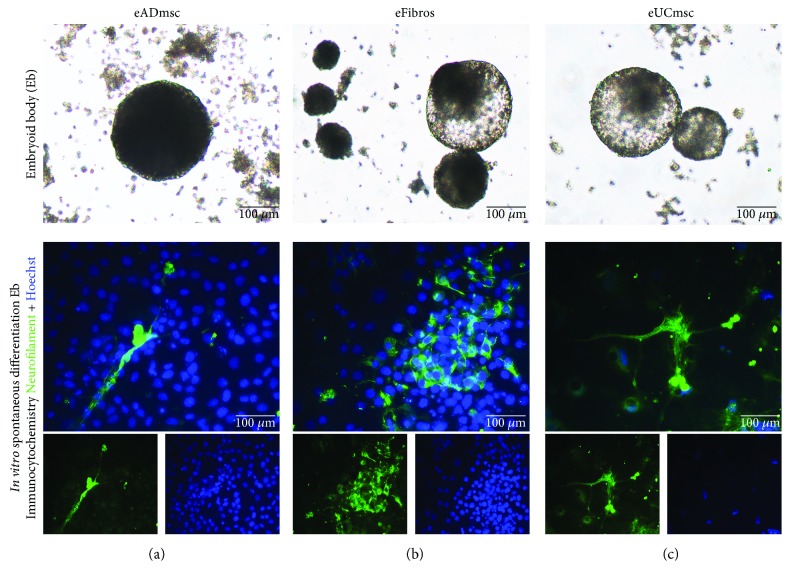
Four-day-old EBs produced from equine iPSCs derived from (a) adipose tissue mesenchymal cells, (b) fibroblasts, and (c) umbilical cord tissue mesenchymal cells. After the spontaneous differentiation of embryoid bodies into multiple lineages, the cells presented a more elongated morphology and were immunocytochemically positive for neurofilament. Merged images of neurofilament/FITC and Hoechst staining: (a) eiPSCs-eADmsc-derived cells, (b) eiPSCs-eFibros-derived cells, and (c) eiPSCs-UCmsc-derived cells, 200x.

eiPSCs-eADmsc, eiPSCs-eFibros, and eiPSCs-eUCmsc were positive for AP (Figures 2(a i), 2(b i), and 2(c i), respectively) and *OCT4* (Figures 2(a ii), 2(b ii), and 2(c ii), respectively) as detected through immunocytochemistry. We were also able to detect higher levels of expression of both *NANOG* (Figure 2(d i)) and *OCT4* (Figure 2(d ii)) after pluripotency induction in all cell lines analyzed. The vector expression was confirmed in eiPSCs between passages 5 and 7, indicating that the exogenous vectors were not silenced until then (Figure 2(d iv)). The eiPSCs were also positive for EB formation (Figures 3(a), 3(b), and 3(c)) and *in vitro* spontaneous differentiation, which was characterized by more elongated morphology and positive neurofilament labeling (Figures 3(a ii), 3(b ii), and 3(c ii)).

### 3.3. Global miRNA Profile Analysis

**Figure 4:**
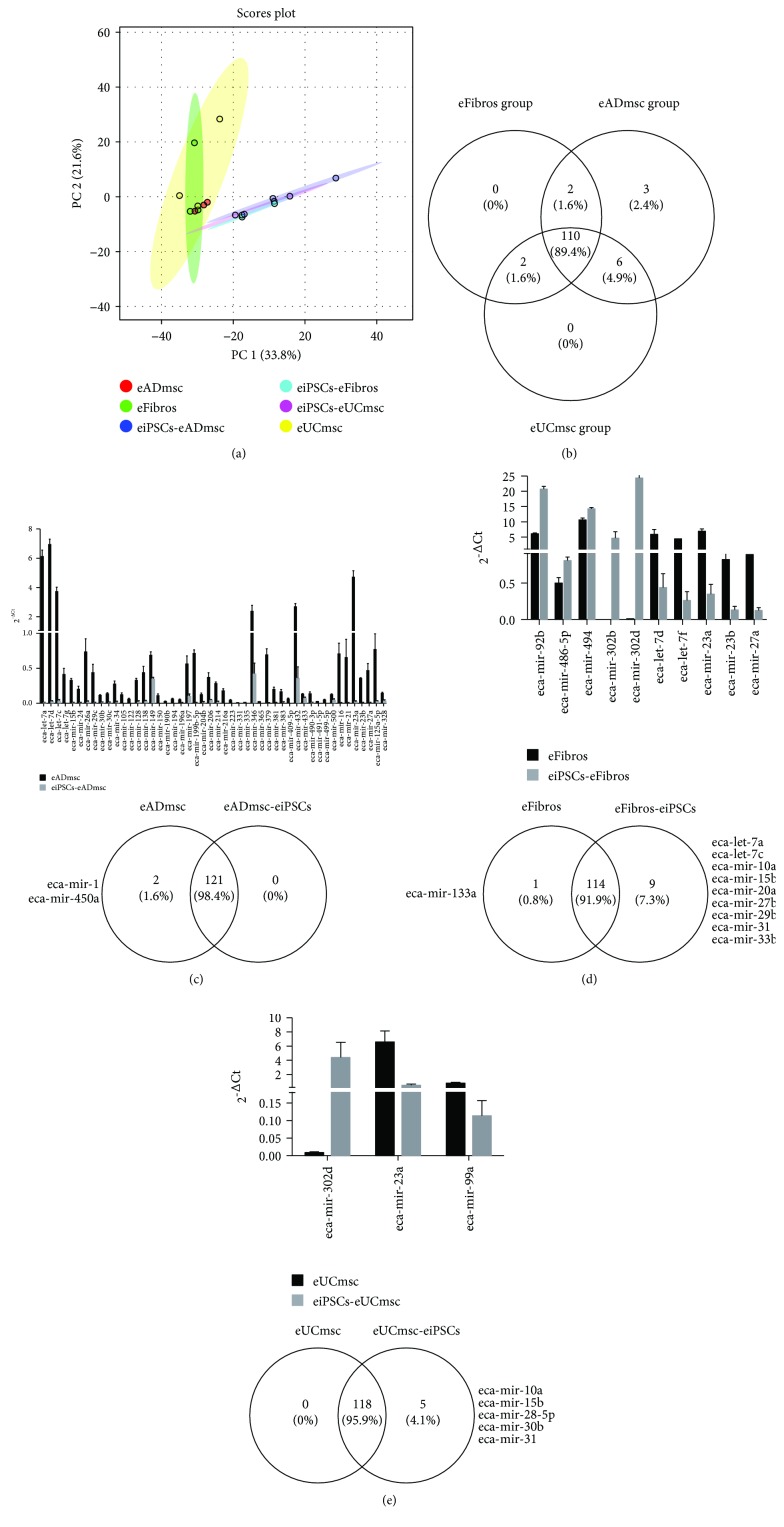
(a) PCA analysis of all 110 miRNAs commonly detected in eiPSCs derived from adipose tissue mesenchymal cells, umbilical cord tissue mesenchymal cells, fibroblasts, and control cells from each of these groups, suggesting slight clustering and difference between the nonreprogrammed and reprogrammed cells. The more clustered and segregated the groups and points, the greater the similarities among the groups. (b) Venn diagram showing distribution of the miRNAs analyzed on the three cell groups and the presence of the 110 miRNAs commonly detected in all of them. (c) Expression levels of miRNA differently expressed between eiPSCs-eADmsc and eADmsc cells (*P* < 0.05) and Venn diagram of miRNAs exclusively expressed in eADmsc and eiPSCs-eADmsc. (d) Expression levels of miRNAs differently expressed between eiPSCs-eFibros and eFibros cells (*P* < 0.05) and Venn diagram of miRNAs exclusively expressed in eFibros and eiPSCs-eFibros. (e) Expression levels of miRNAs differently expressed between eiPSCs-eUCmsc and eUCmsc cells (*P* < 0.05) and Venn diagram of miRNAs exclusively expressed in eUCmsc and eiPSCs-eUCmsc.

The relative levels of 124 equine target miRNAs were analyzed in eADmsc, eUCmsc, and eFibros before and after pluripotency induction. Of these, 123 miRNAs were detected in the eUCmsc group, 124 miRNAs were detected in the eFibros group, and 123 miRNAs were detected in the eADmsc group. Analysis performed with the 110 miRNAs commonly detected for the three groups of cells demonstrated some downregulation of miRNAs in the investigated eiPSCs compared with the levels in the controls (Figures 4(a) and 4(b); Supplemental Figure 11).

By performing a principal component analysis (PCA), despite the relatively high variation in the reprogramming process, we can observe a slight difference and clustering between the eiPSCs and cells prior to reprogramming (Figure 4(a)). Thus, based on miRNA expression and the multivariate analysis approach using PCA analysis, it seems that the cells prior to and after induction present some clustering, although some eiPSCs do not present a similar pattern within cell lines, suggesting heterogeneous cell populations in each group.

#### 3.3.1. Differential miRNA Profile between Fibroblasts and eiPSCs-eFibros

Of the 124 equine miRNAs in the equine fibroblasts prior to and after pluripotency induction, 114 miRNAs were detected in both the control cells and eiPSCs. Of these 114 miRNAs, 10 miRNAs were differentially expressed between the control cells and iPSCs. The miRNAs eca-miR-92b, eca-miR-486-5p, eca-miR-494, eca-miR-302b, and eca-miR-302d were upregulated in the iPSCs compared with their levels in the control cells (Figure 4(d)). Further analysis of the five upregulated miRNAs in the eiPSCs predicted the regulation of 24 signaling pathways (Supplementary material 4), including the regulation of the stem cell pluripotency pathway.

Similarly, eca-let-7d, eca-let-7f, eca-miR-23a, eca-miR-23b, and eca-miR-27a were downregulated in the iPSCs compared with their levels in the control cells (Figure 4(d)). Interestingly, the analysis of the five downregulated miRNAs predicted the regulation of 56 signaling pathways (Supplementary material 5). Among the regulated pathways, stem cell pluripotency was predicted to be regulated by miRNAs differently expressed between the iPSCs and control cell lines from fibroblasts. Interestingly, 41 genes were predicted to be regulated by the upregulated miRNAs in the iPSCs, while 67 genes were predicted to be regulated by the miRNAs upregulated in the control cells. Remarkably, *POU5F* and *MYC* were predicted to be regulated by the miRNAs present in the control cells and not in the eiPSCs. Thus, these results demonstrate that the induction of pluripotency is capable of changing the miRNA levels in eiPSCs derived from eFibros.

We also evaluated the number of miRNAs identified exclusively in each cell group (Figure 4(di)). Of these miRNAs, 608 genes involved in 18 pathways were identified to be regulated by miRNAs exclusively detected in eiPSCs-eFibros, including the regulation of stem cell pluripotency, which is predicted to be regulated by five miRNAs regulating 58 genes. eca-miR-133a, which was detected exclusively in eFibros, regulates 23 genes involved in five pathways (Supplementary material 6).

#### 3.3.2. Differential miRNA Profile between eUCmsc and eiPSCs-eUCmsc

Of the 124 miRNAs analyzed in the equine umbilical cord cells prior to and after pluripotency induction, 118 miRNAs were detected in multiple cell lines, and five miRNAs were detected exclusively in eiPSCs-eUCmsc. Of these 118 miRNAs, three miRNAs were differently detected between control cells and eiPSCs. miRNA-302d was upregulated in the eiPSCs compared with its level in the controls, while miR-23a and miR-99a were downregulated in eiPSCs-eUCmsc compared with their levels in the eUCmsc (Figure 4(e)).

Further analysis of miR-302d predicted the regulation of 11 signaling pathways (Supplementary material 7), whereas the bioinformatics analysis of miR-23a and miR-99a predicted the regulation of 20 signaling pathways (Supplementary material 8). Among the regulated pathways, the regulation of Wnt signaling and fatty acid biosynthesis was predicted to be regulated by miRNAs differently expressed between the iPCSs and control cell lines from the umbilical cord. The analysis of the five miRNAs exclusively detected in eiPSCs-eUCmsc (Figure 4(ei)) predicted the regulation of 35 signaling pathways; among the regulated pathways, the regulation of stem cell pluripotency (Supplementary material 6) is predicted to regulate 38 genes.

#### 3.3.3. Differential miRNA Profile between eADmsc and eiPSCs-eADmsc

Our analysis revealed that 121 miRNAs were shared between eiPSCs-eADmsc and eADmsc, and two miRNAs (eca-miR-1; eca-miR-450a) were detected exclusively in eADmsc (Figure 4(ci)). Those miRNAs are predicted to regulate over 70 gene-related pathways without direct correlation to the mechanisms of pluripotency acquisition or maintenance. Of the 121 miRNAs, 48 miRNAs were differentially expressed between the iPSCs and control cell lines (Figure 4(c)), and these miRNAs were all upregulated in the eADmsc compared with their levels in eiPSCs-eADmsc.

The analysis of the 48 miRNAs predicted the regulation of 68 signaling pathways (Supplementary material 9). Among the predicted pathways, the bioinformatics analysis identified 119 targets within the pathways regulating pluripotency and stem cells (Supplementary material 6). As expected, the miRNAs upregulated in the control cells regulate genes such as SOX2, NANOG, and POU5F and iPSC response genes such as PCGF2, FGF2, TGF3, SKIL, REST, KAT6A, LEFTY2, PAX6, HOXB1, HAND1, NEUROG1, and ISL1. The downregulation of these 48 miRNAs in the eiPSCs indicates that there is decreased control over the expression of pluripotency factors, suggesting efficient reprogramming. Nevertheless, in contrast to the other cell types studied here, the eca-miR-302 family did not express differently in the eADmsc-derived eiPSCs. This miRNA is detected in these cells (Supplementary materials 10 and 11); however, no significant differences were detected probably due to the variation among the cell lines analyzed (Figure 4(c)).

## 4. Discussion

### 4.1. Isolation and Characterization of Equine Mesenchymal Stem Cells

MSCs are known to present specific characteristics, such as adherence to plastic; the capacity for *in vitro* trilineage differentiation under preestablished conditions; the expression of CD44, CD73, CD90, CD105, and MHC I; and the absence of CD34, CD45, CD14 or CD11b, CD79, CD86, CD19, and MHC II expression [19–21, 26]. In this study, equine MSCs and fibroblasts were isolated and they presented plastic adherence competence. Their potential for differentiating into osteogenic, adipogenic, and chondrogenic cells was successfully characterized according to previously described protocols using equine cells [27]. Their immunophenotypic profiles showed high expression of CD44 and MHC I and low expression of CD86 and MHC II. eUCmsc and eBMmsc presented significantly longer doubling time rates than eADmsc and eFibros (*P* < 0.05). Cell proliferation rates are known to vary according to cell origin [28, 29] and donor age [30, 31] and even among horse breeds and individuals [32], which might explain the differences found among the different cell types studied here.

### 4.2. Cellular Reprogramming

Equine iPSCs have already been produced through a PiggyBac-transposon system carrying mouse *OCT4*, *SOX2*, *KLF4*, and *c-MYC* factors (mOSKM) from fetal fibroblasts [6]; adult fibroblasts were induced to pluripotency through Moloney murine leukemia virus vectors using the human *OCT4*, *KLF4*, and *SOX2* genes [7] with the addition of *C-MYC* to produce LIF- (leukemia inhibitory factor-) dependent eiPSCs [9] using mOSKM in foal and adult fibroblasts [8]. Additionally, eiPSCs from filly keratinocytes have been reported [10]. Recently, eiPSCs were produced from eADmsc from a young horse using a TetO inducible lentiviral vector containing mOSKM [11].

Here, we produced eiPSCs derived from adipose tissue mesenchymal cells, umbilical cord tissue mesenchymal cells, and adult equine fibroblasts using a polycistronic excisable lentivirus system [23] containing hOSKM (Table 3). Similar to the equine iPSCs produced by other groups [6–11], the eiPSCs produced here were positive for *OCT4* and *NANOG*. Since *NANOG* expression in this reprogramming system is endogenous, the expression of this transcription factor after pluripotency induction is indicative of the intrinsic reactivation of endogenous pluripotent-related genes.

eiPSCs-eADmsc, eiPSCs-eUCmsc, and eiPSCs-eFibros produced here are positive for AP, EB formation, *in vitro* spontaneous differentiation, and neurofilament labeling after the differentiation of EBs, all of which are significant characteristics of pluripotent cells.

Meanwhile, in contrast to other established eiPSCs [6–11], the cells produced here are exclusively dependent on bFGF, dismissing the need for supplementation with LIF. This characteristic may be related to the reprogramming system, which differs from those previously used.

On the 11th day after reprogramming, eiPSCs-eUCmsc began to present colonies, followed by eADmsc on the 13th day and eFibros on the 15th day. After 20 days in culture, eiPSCs-eADmsc presented the highest number of colonies, followed by eiPSCs-eUCmsc and eiPSCs-eFibros (*P* < 0.05). This differentiated behavior among the cell lines was expected as it has been shown that even after reprogramming, cells may retain a residual epigenetic memory that may affect their capability for reprogramming and differentiation [12], leading us to believe that the cells studied here were subjected to this effect. Considering that eADmsc and eUCmsc are less differentiated than fibroblasts [14], we expected that these cells would present a higher reprogramming rate. Moreover, it has been reported that human fibroblasts present lower reprogramming rates than other lineages [13] as confirmed by Sharma et al. [10], who reported a higher iPSC production efficiency using keratinocytes compared to fibroblasts.

It was not possible to derive eiPSCs from bone marrow mesenchymal cells, and it has been demonstrated that cell reprogramming and pluripotency maintenance are closely related to high cellular proliferation rates [33]; since eBMmsc presented the longest doubling time among the studied cells in this experiment, it was expected that these cells would present a low reprogramming efficiency.

### 4.3. miRNA Profile Analyses

The miRNA profile analyses of the cells in this study showed moderate segregation of eiPSCs-eADmsc, one cell line of eiPSCs-eUCmsc, and eiPSCs-eFibros from the other eiPSCs and control cells (Figure 4(a); Supplemental Figure 11). These results are consistent with the observations made during cell culture and characterization in which eiPSCs-eADmsc responded better to the reprogramming process and eiPSCs-eUCmsc and eiPSCs-eFibros that were grouped with eiPSCs-eADmsc presented the greatest EB formation efficiency in their respective groups, which is indicative of reprogramming. This finding may suggest that the eiPSCs that clustered with the control cells present colonies with more heterogeneous cell populations than the other lineages or might be unevenly reprogrammed. This also affected the segregation between different studied groups in the PCA, making the clustering pattern less pronounced.

Likewise, we observed the presence of miRNAs regulating pluripotency-related genes on cells after pluripotency induction (Figures 4(d) and 4(e)). The members of the miR-302-367 family are known to regulate the levels of pluripotency markers [34], and these miRNAs seem to play a more important role during cellular reprograming than other miRNAs and eca-miR-92. These miRNAs are also increased in ESCs and human iPSCs [16, 17]. Interestingly, the miRNAs from the miR-302/367 family are enhanced in eiPSCs-eFibros and eiPSCs-eUCmsc but not in eiPSCs-eADmsc, probably due to the variation presented in these cells (Supplementary material 10). Furthermore, miR-21, miR-122, miR-128, and the miR-34 family were all predicted to regulate the expression of the OCT4, NANOG, and SOX2 genes, which are increased in eADmsc, in contrast to the findings of eiPSCs-eADmsc. Increased levels of miR-302/367 and the miR-92 family were found in iPSCs and ESCs, while the miRNAs miR-23, miR-27, and miR-30, as well as the let-7 family were increased in the control fibroblasts [16, 17]. Similar expression patterns were found in the eiPSCs studied here and their respective controls. The increased expression of the miRNAs miR-21, miR-23, and miR-206 was found in fibroblasts compared to their levels in porcine iPSCs [15], as we found in our control cells. Moreover, these authors observed that porcine iPSCs with higher expression of the miR-302 family also had a higher reprogramming efficiency [15].

The results found here are consistent with those reported by Porciuncula et al. in 2013 [35], who characterized the reprogramming process to which pluripotency-induced cells were submitted.

Additionally, increases in the miRNAs miR-23 and miR-27 and the let-7 family can be observed in the negative control cells in this study as observed by Wilson et al. in 2009 [16] in their differentiated cells, and a decrease in these miRNAs in eiPSCs is an indication of reprogramming. The overall results of the eiPSC miRNA profile analyses suggest that eiPSCs-eADmsc were more evenly reprogrammed than eiPSCs-eFibros and eiPSCs-eUCmsc.

## 5. Conclusion

In this study, it was possible to isolate and characterize fetal- and adult-derived equine cell lines that were submitted to induced reprogramming *in vitro*. We have shown that it is possible to generate eiPSCs-eADmsc, eiPSCs-eUCmsc, and eiPSCs-eFibros using human reprogramming factors. Under our conditions, it was not possible to generate eiPSCs derived from bone marrow mesenchymal cells, and the use of hOSKM was more efficient than the use of murine factors in the production of equine iPSCs. This conclusion was based on the greater number of colonies found in the cells reprogrammed by hOSKM and the absence of reproducibility in the generation of eiPSCs from mOSKM. Furthermore, the eiPSCs produced in this study are exclusively dependent on bFGF.

The generation of eiPSCs-eADmsc was more efficient than the generation of eiPSCs-eFibros and eiPSCs-eUCmsc. This result corroborated the analysis of the miRNA profiles of the studied cell lines before and after reprogramming, which showed that two eiPSCs-eUCmsc and eiPSCs-eFibros lines' profiles were more similar to their controls than the other generated eiPSCs, which is a possible indication of heterogeneous populations among colonies or even of partially reprogrammed cells. These results are consistent with the hypothesis that the cell types studied in this work might respond differently to the process of reprogramming as shown in the efficiency of induction to pluripotency and the characteristics exhibited by the generated eiPSCs. Notably, further studies aiming at characterizing these cells' pluripotentiality and functionality *in vitro* and *in vivo* are still necessary. Hence, a better understanding of the stable and reproducible reprogramming process constitutes an important step towards clinical trials.

## Figures and Tables

**Table 1:** Specific equine primers used for the detection of endogenous and exogenous transcripts related to pluripotency and the reprogramming vector.

Target	Sequence 5′-3′	Fragment length (bp)	Reference
eOCT4	F: TAGGGTTAGAGCTGCCCCCTC	199	XM_014734675.1
	R: GTTTGTGTTGTCCCTCCCCCA		
eNANOG	F: ACTGCTCATTCAGGACAGCC	200	XM_014740545.1
	R: TCTGCTGGAGGCTGAGGTAT		
eGAPDH	F: GTTTGTGATGGGCGTGAACC	205	NM_001163856.1
	R: ATCGCGCCACATCTTCCC		
hSTEMCCA	F: AAGAGGACTTGTTGCGGAAA	182	Sommer et al., 2009 [23]
	R: GGCATTAAAGCAGCGTATCC		

**Table 2:** Percentage of the mean fluorescence of the surface markers CD44, CD86, MHCI, and MHCII in all equine somatic cells studied. After the flow cytometric analysis, the elevated expression of CD44 and MHCI and decreased expression of CD86 and MHCII were detected. No significantly different results were detected.

	eADmsc (%)	eUCmsc (%)	eBMmsc (%)	eFibros (%)
CD44	92.5 ± 11.9	93.4 ± 5.1	86 ± 15.1	89.7 ± 7.5
CD86	1.8 ± 1.3	1.2 ± 0.7	6.4 ± 5.1	0.6 ± 0.1
MHC I	84 ± 7.5	81.2 ± 17.8	61 ± 4.5	94.13 ± 7.52
MHC II	3 ± 0.5	5.4 ± 1.9	13.2 ± 10	5.7 ± 1.6

**Table 3:** Total number of eiPSC colonies produced and cells lines isolated from eBMmsc, eUCmsc, eADmsc and eFibros using human (hOSKM) and murine (mOSKM) reprogramming factors.

Cell type	Reprogramming factor	Number of colonies observed at p0	Isolated cell lines
eBMmsc	hOSKM	0	0
	mOSKM	0	0
eUCmsc	hOSKM	58	21
	mOSKM	0	0
eADmsc	hOSKM	322	33
	mOSKM	1	1
eFibros	hOSKM	65	30
	mOSKM	0	0

## Data Availability

The data used to support the findings of this study are included within the article.

## Abbreviations

AP: Alkaline phosphatase
bFGF: Basic fibroblast growth factor
BSA: Bovine serum albumin
DNA: Deoxyribonucleic acid
DEPC: Diethyl pyrocarbonate
DT: Mean cell doubling time
eADmsc: Equine adipose tissue mesenchymal cells
EB: Embryoid body
eBMmsc: Equine bone marrow mesenchymal cells
eFibros: Equine adult fibroblasts
eiPSCs: Equine induced pluripotent stem cells
eiPSCs-eADmsc: Equine induced pluripotent stem cells derived from adipose tissue mesenchymal cells
eiPSCs-eFibros: Equine induced pluripotent stem cells derived from adult fibroblasts
eiPSCs-eUCmsc: Equine induced pluripotent stem cells derived from umbilical cord tissue mesenchymal cells
ESCs: Embryonic stem cells
eUCmsc: Equine umbilical cord tissue mesenchymal cells
FBS: Fetal bovine serum
h: Hour(s)
hOSKM: Human *OCT4*, *SOX2*, *KLF4*, and *C-MYC*
IMDM: Iscove's modified Dulbecco's medium
iPSCs: Induced pluripotent stem cells
LIF: Leukemia inhibitor factor
m: Minute(s)
miRNA: MicroRNA
mOSKM: Murine *OCT4*, *SOX2*, *KLF4*, and *C-MYC*
MSCs: Mesenchymal stem cells
PCR: Polymerase chain reaction
PFA: Paraformaldehyde
PBS: Phosphate-buffered saline
RNA: Ribonucleic acid
s: Second(s)

## Supplementary Materials

Supplementary 1 Table S1: primers used for miRNA profile analyses on equine iPSCs. List of the primers used for miRNA profile analysis of equine induced pluripotent stem cells prior to and after reprogramming. List of the primers used for miRNA profile analyses on equine iPSCs.

Supplementary 2 Figure S1: isolation of equine bone marrow mesenchymal cells, characterization by multilineage differentiation, and pluripotency induction. (a) Bone marrow mesenchymal cells, 200x, absence of colonies for eiPSCs colonies on (b) day 18 after pluripotency induction and (b i) on day 31 after pluripotency induction, 200x. (c) Adipogenic differentiated cells were indicated by Sudan black-stained lipid vacuole accumulation, 200x; (d) osteogenic differentiated cells were indicated by calcium deposition, stained with alizarin red, 100x; and (e) chondrogenic differentiated cells were indicated by chondrogenic pellet development, stained with Alcian blue, 100x. Negative control cells maintained the typical spindle-like shape, differently from treated cells. Results were obtained from isolation, characterization by multilineage differentiation, and pluripotency induction of equine bone marrow mesenchymal cells.

Supplementary 3 Figure S2: doubling time assay. The graph shows the doubling time (hours) assay of adipose tissue mesenchymal cells (eADmsc), fibroblasts (eFibros), umbilical cord tissue mesenchymal cells (eUCmsc), and bone marrow mesenchymal cells (eBMmsc). eADmsc and eFibros presented a lower doubling time when compared to eUCmsc and eBMmsc. Different letters indicate significantly different results (*P* < 0.05). Graph showing results of the doubling time assay of equine mesenchymal cells and fibroblasts.

Supplementary 4 Chart S1: pathways regulated by miRNAs increased in eiPSCs-eFibros. List of the pathways regulated by miRNAs increased in eiPSCs derived from fibroblasts.

Supplementary 5 Chart S2: pathways regulated by miRNAs increased in fibroblasts. List of the pathways regulated by miRNAs increased in fibroblasts prior to pluripotency induction.

Supplementary 6 Figure S3: signaling pathways regulating pluripotency of stem cells. Signaling pathways regulating pluripotency of stem cells, regulated by miRNAs found on eiPSCs derived from eADmsc, UCmsc, and eFibros (KEGG PATHWAY Database). Signaling pathways regulating pluripotency of stem cells.

Supplementary 7 Chart S3: pathways regulated by miRNAs increased in eiPSCs-eUCmsc. List of the pathways regulated by miRNAs increased in eiPSCs derived from umbilical cord mesenchymal cells.

Supplementary 8 Chart S4: pathways regulated by miRNAs increased in umbilical cord mesenchymal cells. List of the pathways regulated by miRNAs increased in umbilical cord mesenchymal cells prior to pluripotency induction.

Supplementary 9 Chart S5: pathways regulated by miRNAs increased in adipose tissue mesenchymal cells. List of the pathways regulated by miRNAs increased in adipose tissue mesenchymal cells prior to pluripotency induction.

Supplementary 10 Figure S4: Eca-miR-302 family expression levels on eiPSCs and control cells. The miR-302 family is associated with the maintenance of pluripotency. Although it is not statistically significant in eiPSCs derived from adipose tissue mesenchymal cells, the expression of these miRNAs is detectable in the all eiPSCs studied here. Normalized concentration of the eca-miR-302 family on eiPSCs and control cells.

Supplementary 11 Figure S5: heatmap of the 110 miRNAs commonly detected in eiPSCs and control cells from all three cell types. Heatmap of the miRNAs analyzed among control and eiPSCs, showing clustering of eiPSCs and control groups. Heatmap of the miRNA profile analyzed on control and eiPSCs, showing segregation among eiPSCs and control cells.
