# Supplementary material for: Generation and miRNA Characterization of Equine Induced Pluripotent Stem Cells Derived from Fetal and Adult Multipotent Tissues
Source: Stem Cells Int. 2019 May 2;2019:1393791. doi: 10.1155/2019/1393791 (PMC6525926; doi:10.1155/2019/1393791)
Supplement: Supplementary 1 — Table S1: primers used for miRNA profile analyses on equine iPSCs. List of the primers used for miRNA profile analysis of equine induced pluripotent stem cells prior to and after reprogramming. List of the primers used for miRNA profile analyses on equine iPSCs. [file 1393791.f1.pdf]

Supplemental material 1:
Table S1: Primers used for miRNA profile analyses on equine iPSCs

miRNA	Sequence	Fragment length
eca-let-7a	ugagguaguagguuguauaguu	22
eca-let-7c	ugagguaguagguuguaugguu	22
eca-let-7d	agagguaguagguugcauaguu	22
eca-let-7f	ugagguaguagauuguauaguu	22
eca-let-7g	ugagguaguaguuuguacaguu	22
eca-mir-1	uggaauguaaagaaguauguau	22
eca-mir-10a	uacccuguagauccgaauuugug	23
eca-mir-10b	uacccuguagaaccgaauuugug	23
eca-mir-15b	uagcagcacaucaugguuuaca	22
eca-mir-19a	ugugcaaaucuaugcaaaacuga	23
eca-mir-20a	uaaagugcuuauagugcagguag	23
eca-mir-24	uggcucaguucagcaggaacag	22
eca-mir-25	cauugcacuugucucggucuga	22
eca-mir-26a	uucaaguaauccaggauaggcu	22
eca-mir-27b	uucacaguggcuaaguucugc	21
eca-mir-28-5p	aaggagcucacagucuauugag	22
eca-mir-29b	uagcaccauuugaaaucaguguu	23
eca-mir-29c	uagcaccauuugaaaucgguua	22
eca-mir-30b	uguaaacauccuacacucagcu	22
eca-mir-30c	uguaaacauccuacacucucagc	23
eca-mir-31	aggcaagaugcuggcauagcu	21
eca-mir-33a	gugcauuguaguugcauugca	21
eca-mir-33b	gugcauugcuguugcauugc	20
eca-mir-34	uggcagugucuuagcugguugu	22
eca-mir-92a	uauugcacuugucccggccugu	22
eca-mir-92b	uauugcacucgucccggccucc	22
eca-mir-95	uucaacgggucuuuauugagca	22
eca-mir-96	uuuggcacuagcacauuuuugcu	23
eca-mir-98	ugagguaguaaguuguauuguu	22
eca-mir-99b	cacccguagaaccgaccuugcg	22
eca-mir-105	ucaaaugcucagacuccuguggu	23
eca-mir-106b	uaaagugcugacagugcagau	21
eca-mir-122	uggagugugacaaugguguuug	22
eca-mir-125b	ucccugagacccuaacuuguga	22
eca-mir-127	ucggauccgucugagcuuggcu	22

eca-mir-128	ucacagugaaccggucucuuu	21
eca-mir-130a	cagugcaauguuaaaagggcau	22
eca-mir-130b	cagugcaaugaugaaagggcau	22
eca-mir-132	uaacagucuacagccauggucg	22
eca-mir-133a	uuugguccccuucaaccagcug	22
eca-mir-133b	uuugguccccuucaaccagcua	22
eca-mir-135a	uauggcuuuuuauuccuauguga	23
eca-mir-135b	uauggcuuuucauuccuauguga	23
eca-mir-137	uuauugcuuaagaauacgcguag	23
eca-mir-138	agcugguguugugaaucaggccg	23
eca-mir-141	uaacacugucugguaaagaugg	22
eca-mir-145	guccaguuuucccaggaaucccu	23
eca-mir-148a	ucagugcacuacagaacuuugu	22
eca-mir-148b-3p	ucagugcaucacagaacuuugu	22
eca-mir-149	ucuggcuccgugucuucacuccc	23
eca-mir-150	ucucccaacccuuguaccagug	22
eca-mir-151-5p	ucgaggagcucacagucuagu	21
eca-mir-153	uugcauagucacaaaagugauc	22
eca-mir-155	uuaaugcuaaucgugauaggggu	23
eca-mir-184	uggacggagaacugauaagggu	22
eca-mir-186	caaagaauucuccuuuugggcu	22
eca-mir-187	ucgugucuuguguugcagccgg	22
eca-mir-190	ugauauguuugauauauuaggu	22
eca-mir-190b	ugauauguuugauauugggguu	21
eca-mir-191	caacggaaucccaaaagcagcug	23
eca-mir-193a-5p	ugggucuuugcgggcgagauga	22
eca-mir-194	uguaacagcaacuccaugugga	22
eca-mir-196a	uagguaguuucauguuguuggg	22
eca-mir-197	uucaccaccuucuccacccagc	22
eca-mir-199b-5p	cccaguguuuagacuaucuguuc	23
eca-mir-200c	uaauacugccggguaaugaugga	23
eca-mir-204b	uucccuuugucauccuaugccu	22
eca-mir-205	uccuucauuccaccggagucug	22
eca-mir-206	uccuucauuccaccggagucug	22
eca-mir-208a	auaagacgagcaaaaagcuugu	22
eca-mir-208b	auaagacgaacaaaagguuugu	22
eca-mir-214	acagcaggcacagacaggcagu	22
eca-mir-216a	uaaucucagcuggcaacuguga	22
eca-mir-216b	aaaucucugcaggcaaauguga	22

eca-mir-222	agcuacaucuggcuacugggu	21
eca-mir-223	ugucaguuugucaaauacccca	22
eca-mir-302b	uaagugcuuccauguuuuaguag	23
eca-mir-302c	uaagugcuuccauguuucagugg	23
eca-mir-302d	uaagugcuuccauguuuuagugu__	23
eca-mir-324-5p	cgcauccccuagggcauuggugu	23
eca-mir-328	cuggcccucucugcccuuccgu	22
eca-mir-331	gccccugggccuauccuagaa	21
eca-mir-335	ucaagagcaauaacgaaaaaugu	23
eca-mir-346	ugucugcccgcaugccugccucu	23
eca-mir-361-5p	uuaucagaaucuccagggguac	22
eca-mir-362-5p	aauccuuggaaccuaggugugagu	24
eca-mir-365	uaaugccccuaaaaauccuuau	22
eca-mir-369-3p	aauaauacaugguugaucuuu	21
eca-mir-370	gccugcuggggguggaaccuggu	22
eca-mir-374a	uuauaauacaaccugauaagug	22
eca-mir-374b	auauaauacaaccugcuaagug	22
eca-mir-376a	aucauagaggaaaauccacgu	21
eca-mir-377	aucacacaaaggcaacuuuugu	22
eca-mir-379	ugguagacuauggaacguagg	21
eca-mir-381	uauacaagggcaagcucucugu	22
eca-mir-382	gaaguuguucgugguggauucg	22
eca-mir-383	agaucagaaggugauuguggcu	22
eca-mir-409-5p	agguuacccgagcaacuuugcau	23
eca-mir-410	aauauaacacagauggccugu	21
eca-mir-423-5p	ugaggggcagagagcgagacuuu	23
eca-mir-432	ucuuggaguaggucauugggugg	23
eca-mir-433	aucaugaugggcuccucggugu	22
eca-mir-448	uugcauauguaggaugucccau	22
eca-mir-449a	uggcaguguauuguuagcuggu	22
eca-mir-450a	uuuugcgauguguuccuaauau	22
eca-mir-450b-5p	uuuugcaauauguuccugaaua	22
eca-mir-454	uagugcaauauugcuuauagggu	23
eca-mir-486-5p	uccuguacugagcugccccgag	22
eca-mir-487b	aaucguacagggucauccacuu	22
eca-mir-490-3p	caaccuggaggacuccaugcug	22
eca-mir-491-5p	aguggggaacccuuccaugagg	22
eca-mir-494	ugaaacauacacgggaaaccuc	22
eca-mir-495	aaacaaacauggugcacuucuu	22

eca-mir-499-5p	uuaagacuugcagugauguuu	21
eca-mir-500	uaauccuugcuaccugggugaga	23
eca-mir-502-3p	aaugcaccugggcaaggauuca	22
eca-mir-505	cgucaacacuugcugguuuccu	22
eca-mir-16	uagcagcacguaaauauuggcg	22
eca-mir-21	uagcuuaucagacugauguuga__	22
eca-mir-23a	aucacauugccagggauuucc_	21
eca-mir-23b	aucacauugccagggauuacc__	21
eca-mir-27a	uucacaguggcuaaguuccgc	21
eca-mir-93	caaagugcuguucgugcagguag	23
eca-mir-99a	aacccguagauccgaucuugug	22
eca-mir-125a-5p	ucccugagacccuuuaaccuguga	24

Subtitle: List of the primers used for miRNA profile analysis of equine induced pluripotent stem cells prior and after remogramming.
