# Supplementary material for: Generation and miRNA Characterization of Equine Induced Pluripotent Stem Cells Derived from Fetal and Adult Multipotent Tissues
Source: Stem Cells Int. 2019 May 2;2019:1393791. doi: 10.1155/2019/1393791 (PMC6525926; doi:10.1155/2019/1393791)
Supplement: Supplementary 2 — Figure S1: isolation of equine bone marrow mesenchymal cells, characterization by multilineage differentiation, and pluripotency induction. (a) Bone marrow mesenchymal cells, 200x, absence of colonies for eiPSCs colonies on (b) day 18 after pluripotency induction and (b i) on day 31 after pluripotency induction, 200x. (c) Adipogenic differentiated cells were indicated by Sudan black-stained lipid vacuole accumulation, 200x; (d) osteogenic differentiated cells were indicated by calcium deposition, stained with alizarin red, 100x; and (e) chondrogenic differentiated cells were indicated by chondrogenic pellet development, stained with Alcian blue, 100x. Negative control cells maintained the typical spindle-like shape, differently from treated cells. Results were obtained from isolation, characterization by multilineage differentiation, and pluripotency induction of equine bone marrow mesenchymal cells. [file 1393791.f2.pdf]

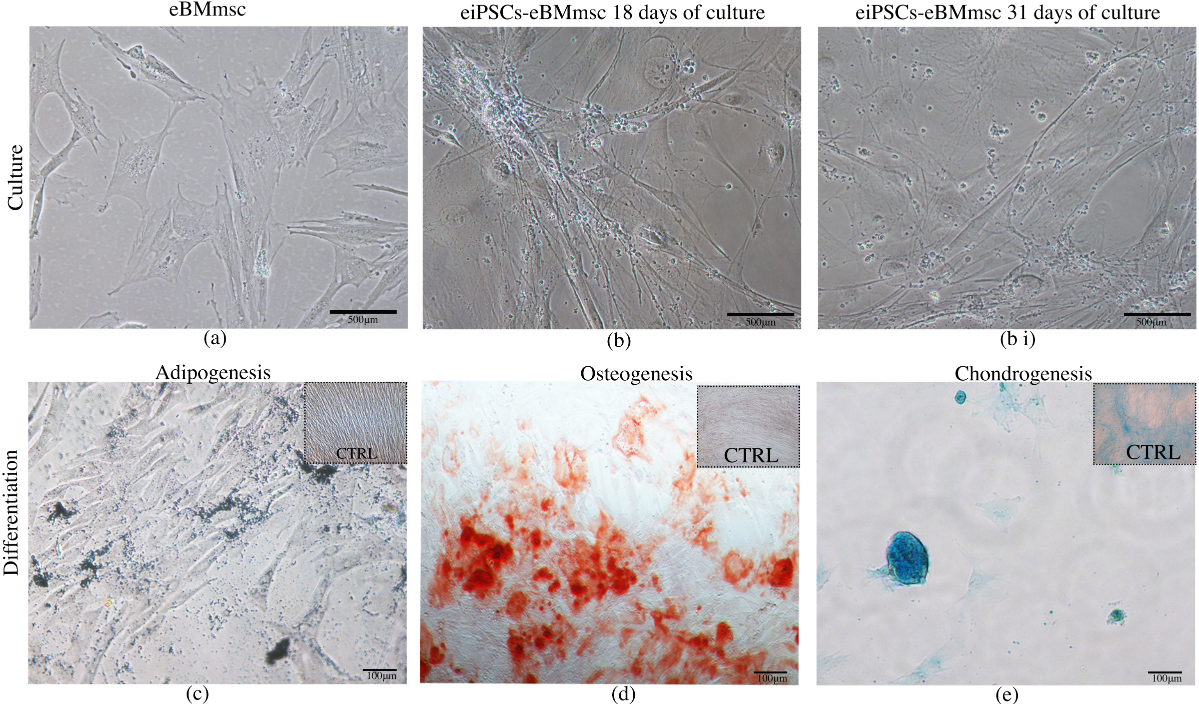
eBMmsc
eiPSCs-eBMmsc 18 days of culture
eiPSCs-eBMmsc 31 days of culture
Culture
500µm
500µm
500µm
(a)
(b)
(b i)
Adipogenesis
Osteogenesis
Chondrogenesis
Differentiation
CTRL
CTRL
CTRL
100µm
100µm
100µm
(c)
(d)
(e)
