# Supplementary figures and images for: Generation and miRNA Characterization of Equine Induced Pluripotent Stem Cells Derived from Fetal and Adult Multipotent Tissues
Source: Stem Cells Int. 2019 May 2;2019:1393791. doi: 10.1155/2019/1393791 (PMC6525926; doi:10.1155/2019/1393791)

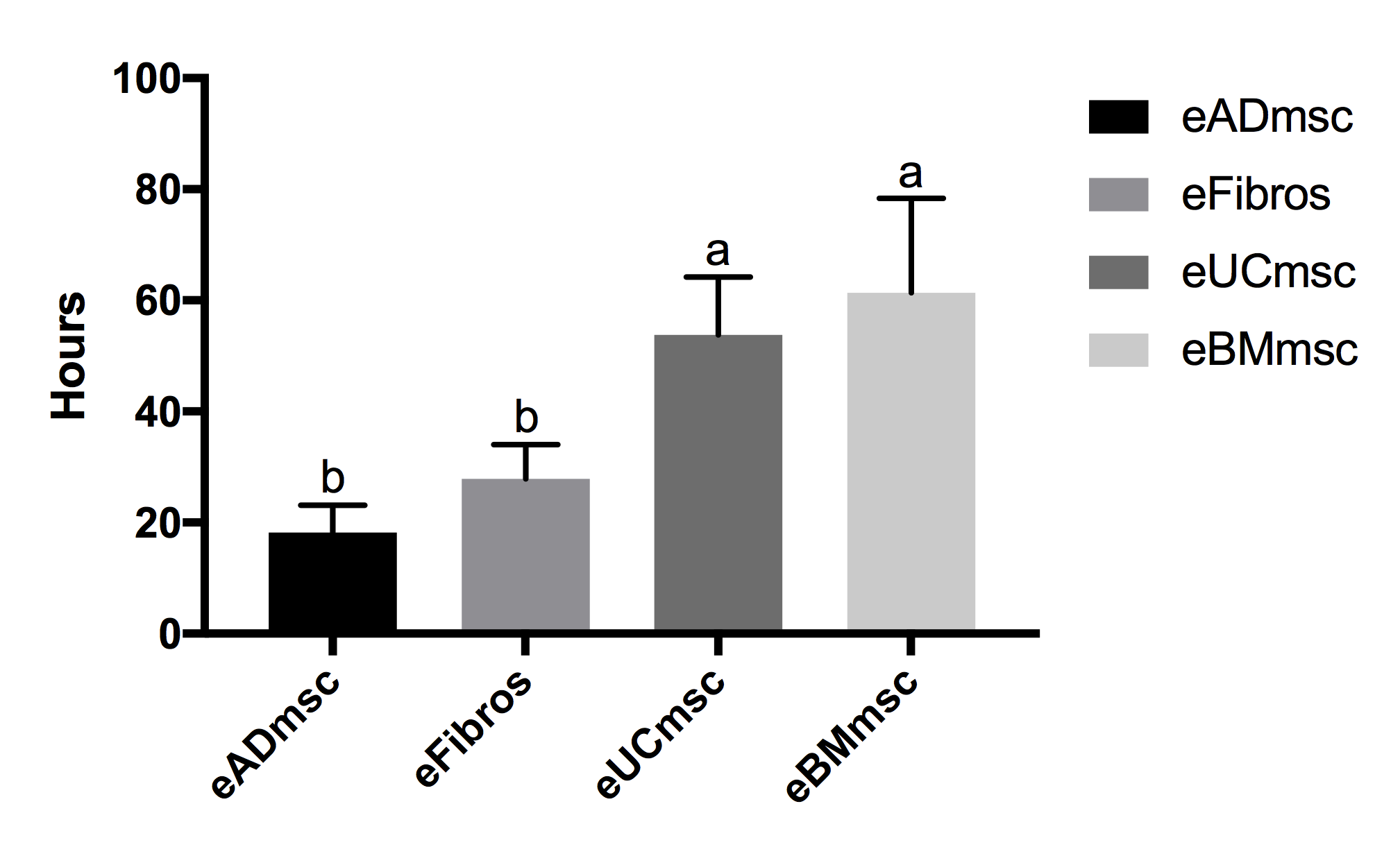

Supplement: Supplementary 3 — Figure S2: doubling time assay. The graph shows the doubling time (hours) assay of adipose tissue mesenchymal cells (eADmsc), fibroblasts (eFibros), umbilical cord tissue mesenchymal cells (eUCmsc), and bone marrow mesenchymal cells (eBMmsc). eADmsc and eFibros presented a lower doubling time when compared to eUCmsc and eBMmsc. Different letters indicate significantly different results (P < 0.05). Graph showing results of the doubling time assay of equine mesenchymal cells and fibroblasts. [file 1393791.f3.tiff]

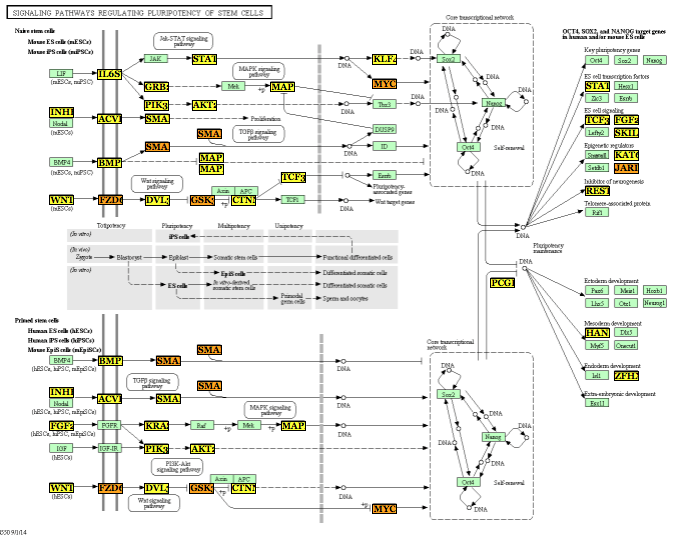

Supplement: Supplementary 6 — Figure S3: signaling pathways regulating pluripotency of stem cells. Signaling pathways regulating pluripotency of stem cells, regulated by miRNAs found on eiPSCs derived from eADmsc, UCmsc, and eFibros (KEGG PATHWAY Database). Signaling pathways regulating pluripotency of stem cells. [file 1393791.f6.png]

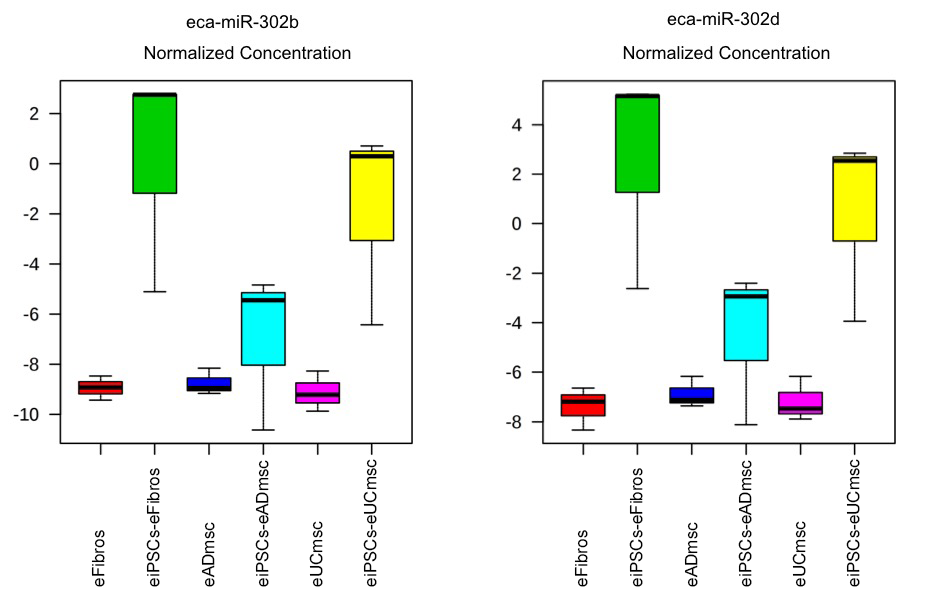

Supplement: Supplementary 10 — Figure S4: Eca-miR-302 family expression levels on eiPSCs and control cells. The miR-302 family is associated with the maintenance of pluripotency. Although it is not statistically significant in eiPSCs derived from adipose tissue mesenchymal cells, the expression of these miRNAs is detectable in the all eiPSCs studied here. Normalized concentration of the eca-miR-302 family on eiPSCs and control cells. [file 1393791.f10.tif]
