# Supplementary material for: Generation and miRNA Characterization of Equine Induced Pluripotent Stem Cells Derived from Fetal and Adult Multipotent Tissues
Source: Stem Cells Int. 2019 May 2;2019:1393791. doi: 10.1155/2019/1393791 (PMC6525926; doi:10.1155/2019/1393791)
Supplement: Supplementary 4 — Chart S1: pathways regulated by miRNAs increased in eiPSCs-eFibros. List of the pathways regulated by miRNAs increased in eiPSCs derived from fibroblasts. [file 1393791.f4.pdf]

Supplemental material 4
Chart S1: pathways regulated by miRNAs increased in eiPSCs-eFibros.

Pathways regulated by miRNAs increased in Fibroblast iPS	Gene number	MiRNAs number
Fatty acid biosynthesis	3	2
Lysine degradation	17	5
Signaling pathways regulating pluripotency of stem cells	41	5
Proteoglycans in cancer	46	5
TGF-beta signaling pathways	25	5
Colorectal cancer	21	5
Fatty acid metabolism	9	3
Oocyte meiosis	27	5
FoxO signaling pathway	36	5
Adherens junction	22	5
Pathways in cancer	71	5
Wnt signaling pathway	33	5
Valine, leucine and isoleucine biosynthesis	2	2
Hippo signaling pathway	38	5
Chronic myeloid leukemia	20	5
Cell clycle	29	5
Endocytosis	39	5
Endometrial cancer	15	5
Non-small cell lung cancer	14	5
Focal adhesion	47	5
Prostae cancer	22	5
Thyroid cancer	8	5
p53 signaling pathway	18	5
Prolactin signaling pathway	17	5
