# Supplementary material for: Generation and miRNA Characterization of Equine Induced Pluripotent Stem Cells Derived from Fetal and Adult Multipotent Tissues
Source: Stem Cells Int. 2019 May 2;2019:1393791. doi: 10.1155/2019/1393791 (PMC6525926; doi:10.1155/2019/1393791)
Supplement: Supplementary 5 — Chart S2: pathways regulated by miRNAs increased in fibroblasts. List of the pathways regulated by miRNAs increased in fibroblasts prior to pluripotency induction. [file 1393791.f5.pdf]

Supplemental material 5

Chart S2: pathways regulated by miRNAs increased in eFibros.

Pathways regulated by miRNAs increased in Fibroblast CTR	Genes number	miRNAs number
Proteoglycans in cancer	111	5
Adherens junction	52	5
TGF-beta signaling pathway	49	5
Hipoo signaling pathway	75	5
Hepatitis B	79	5
Prion diseases	15	5
Viral carcinogenesis	102	5
Lysine degradation	28	5
Glioma	40	5
Thyroid hormone signaling pathway	67	5
Pathways in cancer	189	5
ECM-receptor interaction	34	5
Oocyte meiosis	64	5
Renal cell carcinoma	39	5
Chronic myeloid leukemia	45	5
Cell cycle	70	5
Protein processing in endoplasmic reticulum	87	5
FoxO signaling pathway	75	5
Bacterial invasion of epitelial cells	44	5
Ubiquitin mediated proteolysis	76	5
Transcriptional misregulation in cancer	82	5
AMPK signaling pathway	66	5
Fatty acid biosynthesis	4	5
mTor signaling pathway	38	5
Prostate cancer	51	5
Focal adhesion	103	5
Blader cancer	27	5
Estrogen signaling pathway	49	5
Endocytosis	95	5
Neurotrophin signaling pathway	63	5
Thyroid cancer	18	5
Shigellosis	37	5
Colorectal cancer	34	5
Endometrial cancer	29	5
p53 signaling pathway	38	5
HIF-1 signaling pathway	55	5
Signaling pathways regulating pluripotency of stem cells	67	5
Insulin signaling pathway	69	5
Small cell lung cancer	45	5
Regulation of actin cytoskeleton	93	5
ErbB signaling pathway	45	5
Phosphatidylinositol signaling system	41	5
Pancreatic cancer	36	5
Non-small cell lung cancer	30	5
Wnt signaling pathway	63	5
Sphingolipid signaling pathway	56	5
GnRH signaling pathway	45	5
Inositol phosphate metabolism	28	5
Melanoma	34	5
Acute myelois leukemia	29	5
Central carbono metabolismo in cancer	31	5
Progesterone-mediated oocyte maturation	44	5
HTLV-I signaling pathway	112	5
PI3K-Akt signaling pathway	142	5
TNF signaling pathway	53	5
MAPK signaling pathway	104	5
Apoptosis	38	5
Gap junction	40	5
