# Supplementary material for: Generation and miRNA Characterization of Equine Induced Pluripotent Stem Cells Derived from Fetal and Adult Multipotent Tissues
Source: Stem Cells Int. 2019 May 2;2019:1393791. doi: 10.1155/2019/1393791 (PMC6525926; doi:10.1155/2019/1393791)
Supplement: Supplementary 7 — Chart S3: pathways regulated by miRNAs increased in eiPSCs-eUCmsc. List of the pathways regulated by miRNAs increased in eiPSCs derived from umbilical cord mesenchymal cells. [file 1393791.f7.pdf]

Supplemental material 7

Chart S3: pathways regulated by miRNAs increased in eiPSCs-eUCmsc.

Pathways regulated by miRNA Increased in umbilical iPSCs (eca-mir-302d)	Gene number	MiRNAs number
Lisine degradation	4	1
Wnt signaling pathway	8	1
Mucin type O-Glyan biosynthesis	2	1
Hippo signaling pathway	11	1
Proteoclicans in cancer	8	1
Prolactin signaling pathway	4	1
Oocyte meiosis	5	1
Endocytosis	6	1
Non-small cell lung cancer	2	1
Pathways in cancer	7	1
Colorectal cancer	3	1
