# Supplementary material for: Generation and miRNA Characterization of Equine Induced Pluripotent Stem Cells Derived from Fetal and Adult Multipotent Tissues
Source: Stem Cells Int. 2019 May 2;2019:1393791. doi: 10.1155/2019/1393791 (PMC6525926; doi:10.1155/2019/1393791)
Supplement: Supplementary 8 — Chart S4: pathways regulated by miRNAs increased in umbilical cord mesenchymal cells. List of the pathways regulated by miRNAs increased in umbilical cord mesenchymal cells prior to pluripotency induction. [file 1393791.f8.pdf]

Supplemental material 8

Chart S4: pathways regulated by miRNAs increased in eUCmsc.

Pathways regulated by miRNA Increased in umbilical CTR	Gene number	MiRNAs number
Prion deseases	9	2
Fatty acid biosynthesis	1	1
Proteoglycans in cancer	51	2
Adherens junction	21	2
p53 signaling pathway	23	2
Viral carcinogenesis	45	2
Fatty acid metabolism	7	2
Glycosphingolipid biosynthesis – lacto and neolacto series	4	2
Phosphatidylinositol signaling system	20	2
Glioma	18	2
HIF-1 signaling pathway	28	2
Pathways in cancer	76	2
mTor signaling pathway	18	2
Renal cell carcinoma	17	2
Thyroid hormonesignaling pathway	30	2
Non-small cell lung cancer	15	2
Central carbon metabolism in cancer	16	2
Cell cycle	29	2
Focal adhesion	45	2
Regulation of actin cytoskeleton	44	2
