# Supplementary material for: Generation and miRNA Characterization of Equine Induced Pluripotent Stem Cells Derived from Fetal and Adult Multipotent Tissues
Source: Stem Cells Int. 2019 May 2;2019:1393791. doi: 10.1155/2019/1393791 (PMC6525926; doi:10.1155/2019/1393791)
Supplement: Supplementary 9 — Chart S5: pathways regulated by miRNAs increased in adipose tissue mesenchymal cells. List of the pathways regulated by miRNAs increased in adipose tissue mesenchymal cells prior to pluripotency induction. [file 1393791.f9.pdf]

Supplemental material 9

Chart S5: pathways regulated by miRNAs increased in eADmsc.

Pathways regulated by miRNAs increased in Mesenquimal CTR	Gene number	MiRNAs number
MiRNAs in cancer	155	47
Proteoglycans in cancer	185	47
Pathways in cancer	339	48
Renal cell carcinoma	65	46
Glioma	62	45
ECM-receptor interaction	73	39
TGF-beta signaling pathway	76	45
Hippo signaling pathway	134	45
Hepatitis B	123	46
Pancreatic cancer	63	45
Cell cycle	113	47
Focal adhesion	186	47
Non-small cell lung cancer	52	42
Other types O-glycan biosynthesis	29	29
Transcriptional misregulation in cancer	151	48
Acute myeloid leukemia	55	41
Ubiquitin mediated proteolysis	122	46
Endocytosis	179	44
Protein processing in endoplasmic reticulum	146	43
PI3K-Akt signaling pathway	275	48
Prostae câncer	82	45
Chronic myeloid leucemia	71	44
N-Glycan biosynthesis	45	39
Insulin signaling pathway	124	44
ErbB signaling pathway	78	45
Melanoma	64	44
Adherens junction	68	46
Signaling pathways regulating pluripotency of stem cells	119	45
Thyroid hormone signaling pathway	105	46
Endocmetrial cancer	49	43
Bacterial invasion of epithelial cells	69	45
Thyroid cancer	28	38
Steroid biosynthesis	19	30
p53 signaling pathway	64	43
Neurotrophin signaling pathway	106	44
Phosphatidylinositol signaling pathway	70	43
Axon guidance	106	43
FoxO signaling pathway	117	47
Prion deseases	28	35
Glycosaminoglycan biosynthesis – keratan sulfate	14	25
Small cell lung cancer	75	45
Lysine degradation	45	37
Glycosaminoglycan biosynthesis – chondroitin sulfate/ dermatan sulfate	17	26
Blader cancer	35	41
Colorectal cancer	59	44
Central carbon metabolismo in cancer	59	42
Estrogen signaling pathway	85	45
Spliceosome	109	46
Oocyte meiosis	93	45
Regulation of actin cytoskeleton	173	46
Circadian rhythm	29	31
mTor signaling pathway	56	44
Progesterone mediated oocyte maturation	76	44
RNA transport	139	47
Ras signaling pathway	181	46
Long-term potentation	58	42
Salmonella infection	67	41
Sphingolipid signaling pathway	100	45
Rap1 signaling pathway	169	46
Prolactin signaling pathway	61	44
Inositol phosphate metabolismo	53	41
Fatty acid elongation	20	30
Gap juntion	72	42
Wnt signaling pathway	116	45
Dorso-ventral axis formation	25	38
Ribosome	111	39
